# Supplementary material for: The genome sequence of the Grey Poplar Bell, Epinotia nisella (Clerck, 1759)
Source: Wellcome Open Res. 2023 Jun 28;8:281. [Version 1] doi: 10.12688/wellcomeopenres.19621.1 (PMC11109716; doi:10.12688/wellcomeopenres.19621.1)
Supplement: Peer review history

# Reviewer report: Reviewer response for version 1

**DOI:** 10.21956/wellcomeopenres.21737.r71154

**Published:** 2024-05-13

**Authors:** Jerome H L Hui

**Affiliations:** 1 Chinese University of Hong Kong, Hong Kong, China

Boyes and colleagues report the genome sequence of a male grey poplar bell *Epinotia nisella *(Clerck 1759). This species is widely distributed in Britain. Molecular data of this species are scarce prior to this report, and are all COI sequences deposited to the NCBI database. This new genome resource will be very useful for further studies, such as understanding their geographical distribution, population structure, evolutionary relationships with other lepidopterans, and the underlying mechanism contributing to their (morphological) polymorphism.

 This genome resource is excellent from the summary statistics, with high BUSCO numbers and sequence continuity (scaffold N50), and majority of sequences contained on the 28 pseudochromosomes (plus mitochondrion). To sum up, this is a valuable contribution.

Are sufficient details of methods and materials provided to allow replication by others?

Yes

Is the rationale for creating the dataset(s) clearly described?

Yes

Are the datasets clearly presented in a useable and accessible format?

Yes

Are the protocols appropriate and is the work technically sound?

Yes

Reviewer Expertise:

Genomics, evolution, invertebrates

I confirm that I have read this submission and believe that I have an appropriate level of expertise to confirm that it is of an acceptable scientific standard.

# Reviewer report: Reviewer response for version 1

**DOI:** 10.21956/wellcomeopenres.21737.r77044

**Published:** 2024-05-11

**Authors:** Sivasankaran Kuppusamy

**Affiliations:** 1 Division of Taxonomy and Biodiversity, Entomology Research Institute, Loyola College, Chennai, Tamil Nadu, India

The authors have done the whole genome sequence of *Epinotia nisella *(Clerck, 1759). The authors sequenced over 585.0 million base pairs for the genome assembly using 37 scaffolds. The authors observed 19, 249 gene transcripts and 18,952 protein-coding genes during genome assembly. The standard methods were used for DNA isolation and followed appropriate software for the sequence assembly and annotations.  

 Overall, there are corrections on this manuscript. The manuscript is accepted scientific standards.

Are sufficient details of methods and materials provided to allow replication by others?

Yes

Is the rationale for creating the dataset(s) clearly described?

Yes

Are the datasets clearly presented in a useable and accessible format?

Yes

Are the protocols appropriate and is the work technically sound?

Yes

Reviewer Expertise:

Phylogenetic analysis of moths

I confirm that I have read this submission and believe that I have an appropriate level of expertise to confirm that it is of an acceptable scientific standard.

# Reviewer report: Reviewer response for version 1

**DOI:** 10.21956/wellcomeopenres.21737.r74980

**Published:** 2024-03-04

**Authors:** Ann-Marie Waldvogel

**Affiliations:** 1 University of Cologne, Cologne, Germany

This report presents a high-quality genome assembly of the lepidoptera genome of *Epinotia nisella*, a species that is of scientific interest for its variation in wing pattern coloration. With this, the rational for the generation of high-quality genomic resources is obvious and the genome assembly with furthermore contribute to comparative studies. 

 The assembly has been generated and curated according to standard state-of-the art methodology and fulfills all expected criteria by its summary statistics.

 Here are some minor comments:

 *Lepidopterans are known to be very beautiful, and I highly recommend adding a nicer picture that documents the coloration more clearly.

 *Last paragraph of the description of background: 

 "A genome ... will contribute to our **understanding** of the genomic basis of **morphotype **polymorphism."

Are sufficient details of methods and materials provided to allow replication by others?

Yes

Is the rationale for creating the dataset(s) clearly described?

Yes

Are the datasets clearly presented in a useable and accessible format?

Yes

Are the protocols appropriate and is the work technically sound?

Yes

Reviewer Expertise:

ecological genomics, biodiversity genomics

I confirm that I have read this submission and believe that I have an appropriate level of expertise to confirm that it is of an acceptable scientific standard.
